# Medial patellofemoral ligament reconstruction using a bone groove and a suture anchor at patellar: a safe and firm fixation technique and 3-year follow-up study

**DOI:** 10.1186/s13018-016-0473-z

**Published:** 2016-11-14

**Authors:** Hong-De Wang, Jiang-Tao Dong, Shi-Jun Gao

**Affiliations:** Department of Orthopedics, Third Hospital of Hebei Medical University, 139 Ziqiang Road, Shijiazhuang, 050051 Hebei People’s Republic of China

**Keywords:** Medial patellofemoral ligament reconstruction, Combined bone groove and suture anchor fixation, Safety angle

## Abstract

**Background:**

Graft fixation is critical to the restoration of the medial patella of femoral ligament function and long-term success. Numerous fixations at the patella have been described, while the complications including patellar fractures, violation of the posterior patella and delay of tendon-to-bone healing remain significant challenges. Here, we describe a safe and firm fixation at the patellar for medial patellofemoral ligament (MPFL) reconstruction and explore the safety angle of drilling the suture anchor at different morphology of the patellar. Moreover, we evaluate the results at a 3-year follow-up.

**Methods:**

Combined bone groove and suture anchor fixation at the patella was performed on 26 patients (16 females, 10 males; mean age 26.3 ± 4.7 years) diagnosed with recurrent patellar dislocation. The drilling direction of the suture anchor referred to the safety angle according to the Wiberg type classification. The safety angle was defined as the angle between the drill tunnel and a line that connected the medial and lateral margins of the patella and was established following computed tomography assessment of 117 patients who were diagnosed with patellar dislocation in our hospital according to the Wiberg type classification (I:29, II:65, III:23). X-ray, Lysholm, Kujala and Tegner scores were obtained preoperatively and at the time of final follow-up.

**Results:**

There were no patellar complications, including fracture and redislocation. Average congruence, patella tilt angles and lateral patella angle were significantly changed (*P* < 0.01). The Lysholm, Kujala and Tegner scores were significantly increased (*P* < 0.01). The safe angles of male and female patients according to the patellar Wiberg type classification were less than 45.32 ± 1.76 and 41.20 ± 1.33, 69.74 ± 1.38 and 63.66 ± 1.45 and 84.11 ± 1.67 and 80.26 ± 1.73, respectively.

**Conclusions:**

We achieved encouraging results with this fixation at the patellar. When drilling from Wiberg type I to type III patellar, the suture anchor should be more vertical. When fixing the patellar of female patients, the drilling suture anchor should be more sloping.

## Background

Patellar dislocation is primarily an injury of younger, female patients [1]. The medial patellofemoral ligament (MPFL) is viewed as the most important static structure in preventing the lateral dislocation of the patella [2, 3] and provides 57–63% of the patella’s medial soft tissue restraint [4, 5]. Consequently, the MPFL is ruptured in a high percentage of patellar dislocations [6–8]. As it is understood that the rupture of the MPFL is the main pathoanatomy of patellar dislocations, loosening of the restraint of the MPFL may lead to patellar dislocation occurring frequently. Conservation treatment cannot restore the function and anatomical structure. So MPFL reconstruction has become a widespread surgical technique for reconstruction of patellofemoral stability.

During MPFL reconstruction, graft fixation is critical to the restoration of MPFL function and long-term success. A number of techniques have been used to fix the graft to the patellar MPFL attachment site, including the patellar bone tunnel (PBT) [9–11] and patellar suture anchor (PSA) techniques [9, 12–14]. Few studies have mentioned the definite fixation site at the patellar, and patellar fractures [9, 15], violation of the posterior patella and delay of tendon-to-bone healing remain significant challenges. Although Jia et al. used a bone-fascia tunnel at the medial margin alone, decreasing the risk of patellar fracture comparing with creating a bone tunnel, the micro-motion of the graft in the bone-fascia tunnel occurs during knee flexion-extension, increasing the risk of delayed or insufficient tendon-to-bone healing [16]. Philip et al. described their technique of fixing the graft at the medial margin using two suture anchors. Although the fixation strength is good for tendon-bone healing, the bone bridge between the two suture anchors and the weakness of the media margin increases the risk of patellar fracture [17]. Additionally, the morphology of the patellar is different, so the drilling direction of the suture anchor is not constant [17].

Here, we describe a safe and firm fixation at the patellar in MPFL reconstruction and explore the safety angle of fixing the suture anchor. We hypothesized that combined bone groove and suture anchor fixation at the patella MPFL attachment site would achieve satisfactory restoration of patellar stability and significant knee function improvement. The safety angle provides a safe guide for placing the suture anchor at different morphology of the patellar.

## Patients and methods

### Patients

Twenty-six patients diagnosed with recurrent lateral patella dislocations underwent MPFL reconstruction using our novel approach by one surgeon (SG) from January 2010 to January 2013. This study was approved by the ethics committee of the Third Hospital of Hebei Medical University, and all the patients provided written informed consent. Patient demographics are shown in Table 1. Due to loosening of the restraint of the MPFL, all patients did not improve with conservative treatment. The indication for MPFL reconstruction was symptomatic patients who had experienced at least two lateral patellar dislocations. These lateral patellar dislocations were diagnosed by history taking, physical examination, X-rays, computed tomography (CT) scans and magnetic resonance imaging (MRI).

**Table 1:** Patient demographics

Demographic data	Value
No. of cases	26
Gender (F/M)	16/10
Side (L/R)	15/11
Age at operation (year)	26.3 ± 4.7
Follow-up periods (month)	38.2 ± 3.6
*Q*-angle	13.2 ± 2.3
TT–TG distance (mm)	14.2 ± 1.6

Exclusion criteria are as follows: (1) *Q*-angle >20° in female patients and >17° in male patients [18], (2) patellar height (Insall-Salvati) index >1.2 [19], (3) the horizontal distance between the tibial tubercle and the centre of the trochlear groove (TT–TG) >20 mm [20], (4) Outerbridge grades III and IV patellofemoral articular cartilage degeneration [21], (5) severe trochlear dysplasia, (6) Wiberg grades IV and V patella dysplasia, (7) multiple knee ligament injuries requiring surgery and (8) previous surgery on the injured knee.

To obtain the safety angle of fixing the suture anchor, we recruited 117 patients who were diagnosed with patellar dislocation in our hospital from 2010 to 2013 according to the Wiberg type classification. There were 29 cases of type I, 65 cases of type II and 23 cases of type III. We used picture archiving and communications system (PACS) to measure the safety angle of fixing the suture anchor.

### Surgical technique

#### Intra-articular inspection and lateral reticular release

Physical examination of the operative knee was performed under spinal anaesthesia to assess the degree of patellar instability. Arthroscopy was first performed to evaluate the intra-articular cartilage, which included diagnosing chondral lesions and evaluating the menisci, ligaments and patellar tracking. Surgical treatment of all abnormal conditions, such as repairing the injury of cartilage, meniscectomy or suture for meniscus tear, resection of pathologic plica, and taking out loose body, was performed if necessary. A lateral retinacular release was performed arthroscopically with a banana knife on those patients with moderate patellar maltracking or tight lateral structures.

#### Harvesting and preparing the gracilis tendon

The gracilis tendon was harvested through a 2- to 3-cm vertical incision over the pes anserinus. The anserinus bursa was opened, and the gracilis tendon was exposed and released with a stripper. The gracilis tendon was used as an autograft. The tendon should be at least 18 cm after removing all muscle tissue. The diameter of the folded graft should be equal or greater than 7 mm as measured with a sizing guide. This procedure is necessary to define the diameter of the femoral tunnel and absorbable interference screw. The tendon was sutured with no. 2 non-absorbable braided suture (Arthrex Inc., Naples, Florida, USA) with a 2-cm length at both free ends.

#### Preparation of the patellar bone groove and suture anchor fixation

**Fig. 1:**
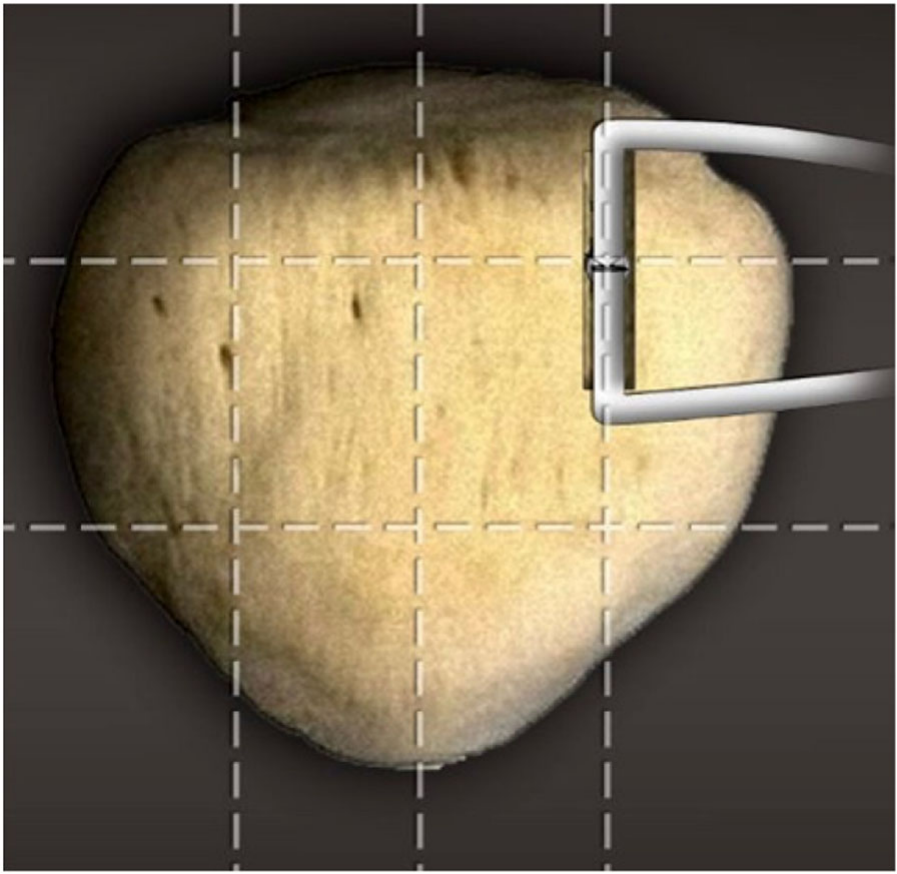
Combined bone groove and suture anchor patella fixation. The groove was created at the upper one third and medial one fourth of the patellar surface

**Fig. 2:**
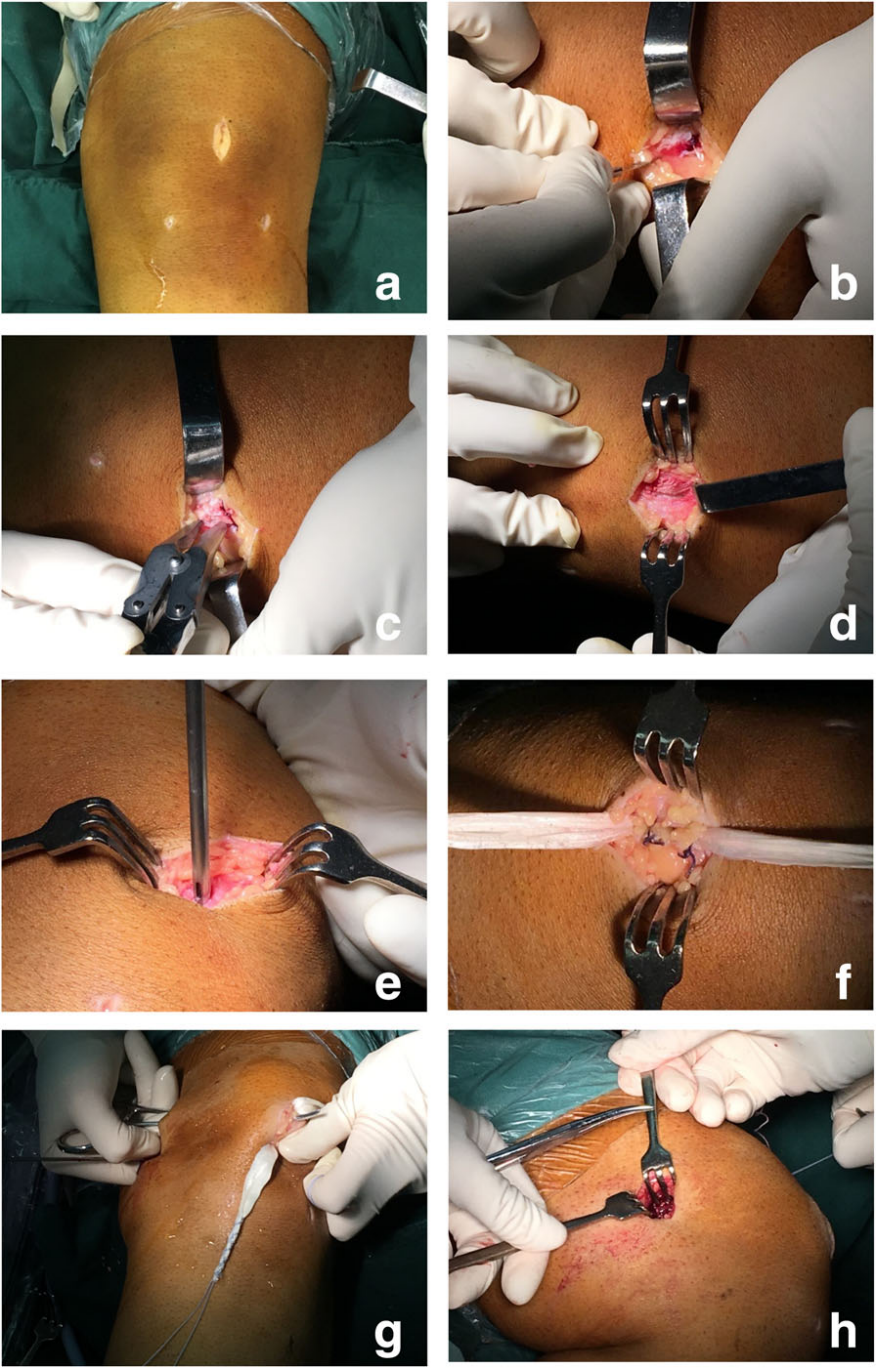
**a** A longitudinal 2.5-cm incision was made in the proximal third of the patella. **b** The deep fascia and periosteum is incised with an approximately 2.5-cm longitudinal cut. **c** Creating a bone groove with a rongeur at the upper one third and medial one fourth of the patellar surface. **d** The patellar bone groove was created. **e** A 3.0-mm-diameter suture anchor is used to strengthen the fixation of the graft. The site of drilling was in the middle of bone groove. The direction of the drilling referred to the safety angle according to patellar morphology. **f** The sutures on the anchor were then tied around the graft. The graft was then embedded into the periosteum and deep fascia with absorbable suture. **g** The two free ends of the graft were pulled out through the subcutaneous fascial layers. **h** The two free ends were pulled into the femoral tunnel by pulling the two sutures

**Fig. 3:**
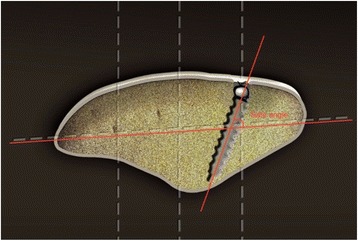
The *safe angle* was defined as the angle between the drilling tunnel and the connecting line. *Lines* connecting the upper-third horizontal plane and the medial and lateral margins of the patella were created on CT scans. The connecting line was divided into four parts. The area representing the medial one fourth of the patella surface was marked as the drilling site. The direction of the drilling referred to the safety angle according to patellar morphology

The preparation of the patellar groove was performed with the knee extended. A longitudinal 2.5-cm incision was made into the proximal third of the patella (Fig. 2a). The deep fascia and periosteum was incised longitudinally. The periosteum was then carefully dissected so as to retain it completely. The bone groove (2-cm length, 4–5-mm width, 3-mm depth) was created with a rongeur at the upper one third and medial one fourth of patellar surface (Figs. 1 and 2b–d). Only one 3.0-mm-diameter suture anchor (BioComposite SutureTak Suture Anchors, Arthrex Inc.) was used to strengthen graft fixation (Fig. 2e) in the middle of the bone groove. To place the suture anchor, the 2.8-mm-diameter drill guide for the suture anchor was used to create a pilot hole in the middle of bone groove. The direction of the drilling referred to the safety angle according to patellar morphology (Fig. 3, Table 3), in case the suture anchor passed through the patella. Tension was applied to the suture to confirm purchase within the patella. The middle of the graft was placed into the bone groove. The sutures on the anchor were then tied around the graft. The graft was embedded by suturing it to the periosteum and deep fascia with absorbable suture (Fig. 2f).

#### Identification of the femoral attachment and regulation of graft tension

**Fig. 4:**
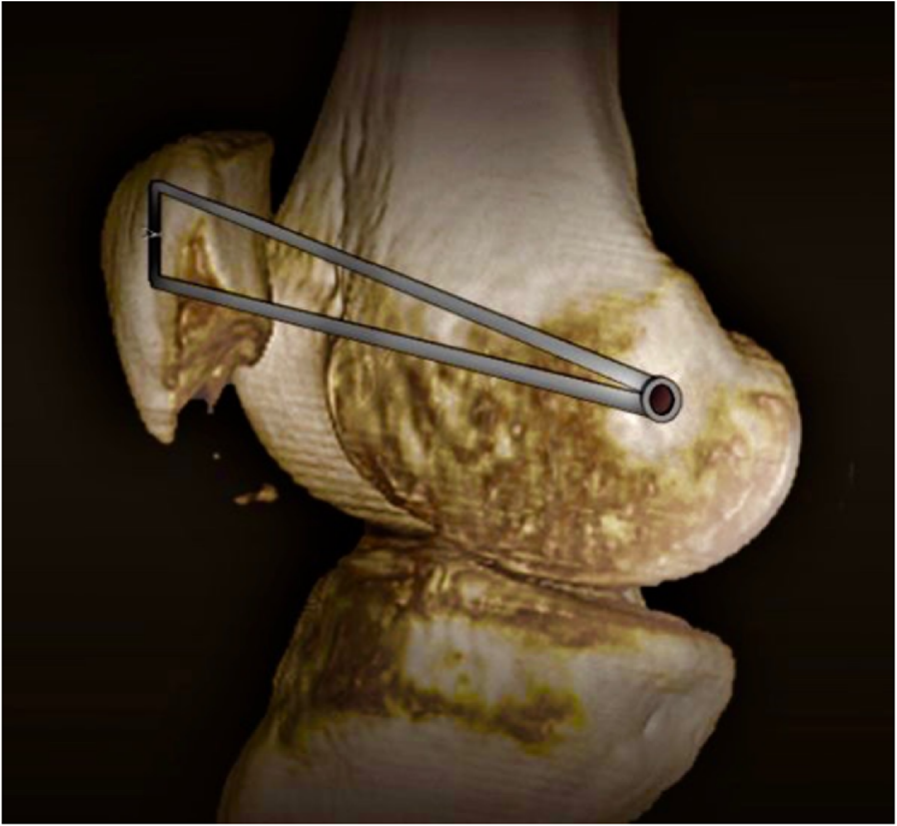
Our safe and firm approach is performed on the patellar and femoral attachments

The anatomic femoral insertion of the MPFL, which is distal to the adductor tubercle and proximal to the medial epicondyle, was palpated [22]. A 2-cm longitudinal incision was made in this area, and subcutaneous tissue was dissected to expose the cortical bone. Care should be taken to avoid injuring the infra-patellar branch of the saphenous nerve. A guide pin with an eyelet was placed in this area, and drilling was performed in an antero-superior direction under fluoroscopic guidance. The femoral tunnel was then drilled using a cannulated reamer. The diameter of the femoral tunnel depends on the diameter of the two-bundle graft. The two free ends of the graft were passed through with two no. 2 absorbable sutures. Then the two free ends were pulled out through the subcutaneous fascial layers and pulled into the femoral tunnel by pulling the two sutures (Fig. 2g, h). The sutures were kept under tension during this procedure. Graft tension and patellar tracking were evaluated arthroscopically throughout the normal range of motion. The tension of each bundle was regulated to avoid rotation or dislocation. The two free ends were fixed with an absorbable interference screw (MILAGRO, DePuy Mitek, Warsaw, Indiana, USA) in 30° of knee flexion (Fig. 4). Patellar tracking was confirmed, and no suture anchors were passed through the patella arthroscopically. The wound was closed in layers.

#### Postoperative rehabilitation

The knee was immobilized with a knee brace in an extended position for 6–8 weeks. To achieve the goal of a 90° flexion by 6 weeks, continuous passive motion was performed over a range of 0–30° twice a day. Partial weight-bearing was permitted after 6 weeks. Full weight-bearing and full range of motion were permitted after 8 weeks. During the eight postoperative weeks, patients wore the knee brace after a range of motion exercises. Quadriceps setting and ankle pumping exercises were performed daily to promote muscle recovery. Mild sports were permitted 3 months postoperatively. All sports were permitted 6 months postoperatively.

#### Outcome evaluation

Lysholm, Kujala and Tegner scores were obtained before and after surgery. All patients had radiographs of their knees taken before surgery and at the time of final follow-up. A CT scan of the patient’s knee was used to calculate the congruence angle, lateral patellofemoral angle and patellar tilt angle. The median follow-up period was 38.2 months (range 35–42 months).

#### Determining safe angle and suture anchor position with computed tomography

Normal patellas were used to calculate the safe angle of this technique using the picture archiving and communications system (PACS) of the Third Hospital of Hebei Medical University. The safe angle was defined as the angle between the drilling tunnel and the connecting line (Fig. 3). Lines connecting the upper-third horizontal plane and the medial and lateral margins of the patella were created on CT scans. The connecting line was divided into four parts. The area representing the medial one fourth of the patella surface was marked as the drilling site. The safe angle was measured by the first author. The safe angle was measured using a Siemens Somatom Definition AS (Siemens Healthcare GmbH Henkestr. 127, 91052 Erlangen, Germany) with a scan time of 0.5 s and 2.5-mm-slice thickness. The X-ray tube voltage was 120 kV with a scan exposure of 250 mA. Each subject was examined in a supine position during CT examination.

#### Statistical analysis

Statistical analysis was performed using SPSS 20.0 (IBM, Armonk, NY, USA). *t* tests were used to compare safety angles. Wilcoxon signed-rank tests were used to compare Lysholm, Kujala and Tegner scores, Insall-Salvati ratio, congruence angle, lateral patellofemoral angle, patellar tilt angle and safety angles. A *P* value <0.05 was defined as significant. All data are presented in the form of mean ± standard deviation.

## Results

Of the 26 patents included in this study, all achieved a full range of motion. No patients suffered from complications, including patellar fracture and redislocation. The safe angles of male and female patients according to the patellar Wiberg type classification were less than 45.32 ± 1.76 and 41.20 ± 1.33, 69.74 ± 1.38 and 63.66 ± 1.45 and 84.11 ± 1.67 and 80.26 ± 1.73 (Table 3). The congruence angle significantly decreased from 19.6 ± 5.7 to 6.1 ± 2.3. The lateral patella angle changed from −7.2 ± 3.3 before surgery to 5.6 ± 1.3 postoperatively, which was significant. The patellar title angle was significantly decreased from 22.3 ± 4.5 to 13.2 ± 3.7 (Table 2). At final follow-up, the Lysholm score was improved from an average of 59.6 ± 4.6 before surgery to 90.3 ± 3.2 postoperatively, the Kujala score was improved from 53.2 ± 8.3 to 89.4 ± 7.6 postoperatively and the Tegner score improved from 3.1 ± 1.2 to 6.2 ± 2.5 postoperatively (*P* < 0.01, Table 2).

**Table 2:** Knee function and radiographic measurements

	Preparation	Postoperation	*P* value
Lysholm score	59.6 ± 4.6	90.3 ± 3.2	<0.01
Kujala score	53.2 ± 8.3	89.4 ± 7.6	<0.01
Tegner score	3.1 ± 1.2	6.2 ± 2.5	<0.01
Congruence angle	19.6 ± 5.7	6.1 ± 2.3	<0.01
Lateral patella angle	−7.2 ± 3.3	5.6 ± 1.3	<0.01
Patellar tilt angle	22.3 ± 4.5	13.2 ± 3.7	<0.01

## Discussion

This study had three important findings. First, using combined bone groove and suture anchor fixation at the patellar MPFL attachment site is a safe and firm fixation, decreasing the risk of patella fracture. The safe angle for placing the suture anchor provides surgeons with a safe and easy guide for fixing the suture anchor at the morphology of the patellar. Second, this technique is good for tendon-bone healing. Finally, this technique is more similar to anatomic MPFL reconstruction.

To date, there were more than 100 different MPFL reconstruction techniques proposed [9–12, 14, 23], mainly using the PBT [9–11] or PSA [12–14, 23] techniques. A patellar fracture is a major complication. Drilling from the medial border of the patella in a lateral direction is a commonly noted characteristic of fracture cases because of the weakness of the medial border. So creating a bone groove on the anterior cortex decreases the risk of medial border fracture compared with any other technique. Anatomic study confirmed [22] that the MPFL patellar insertion mostly occurs though an aponeurosis composed of the tendons of the vastus medialis obliquus (VMO) and the vastus intermedius (VI). This aponeurosis guides the patella in the trochlea during range of motion, making it very important for the integrity of MPFL as a stabilizer. Many authors discuss the continuity between the VMO and VI [24, 25]. Our technique minimizes damage to this continuity. Fracture of the patella by using PBT increased bone loss resulting from using a tunnel technique to pass the tendon graft through the patella [26, 27]. The fracture of the bone bridge between the two tunnels increases the risk of fixation failure and weakens the patella, as reported by some authors that the medial bone bridge fractured in the X-ray [15, 28, 29]. Creating a bone groove can significantly decrease the bone loss and avoid the bone bridge fracture. Although Philip et al. described their technique of fixing the graft at the medial margin using two suture anchors and achieved satisfied outcomes[17], using two suture anchors has also been reported to be complicated by patella fractures [30]. These fractures were because of the disruption of the blood supply to the patella [26].Using only one suture anchor performed in this technique has little influence on the blood supply of the medial patella. The risk of patella fracture is therefore effectively reduced. No fractures occurred in the 26 cases that received our MPFL reconstruction technique.

**Fig. 5:**
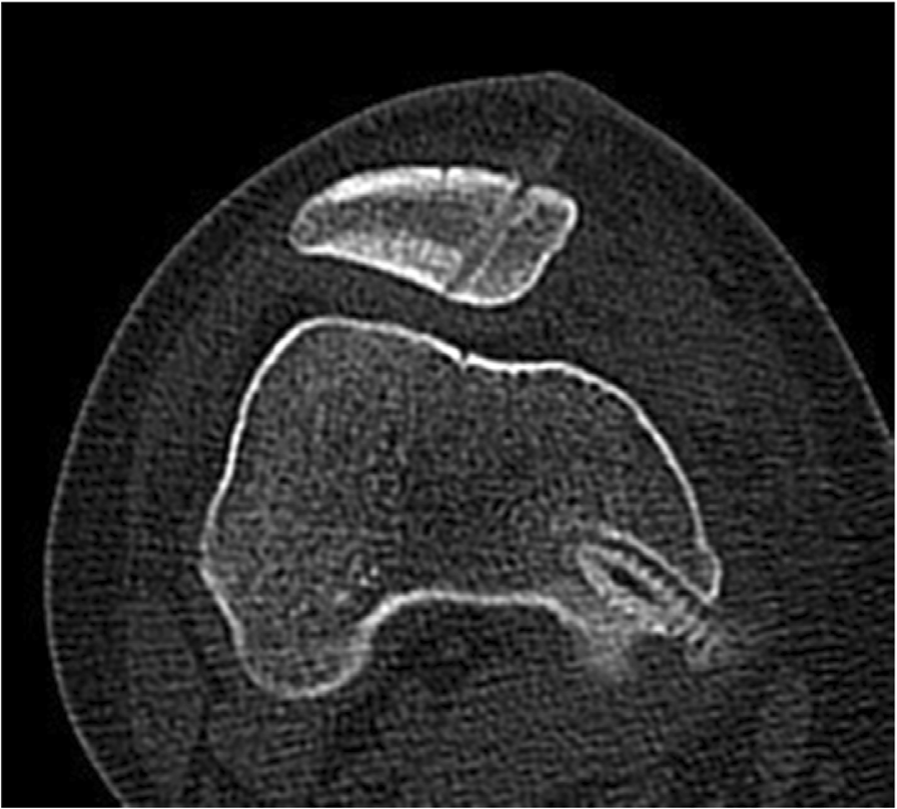
The suture anchor passing through the patella, as shown on CT scans

**Fig. 6:**
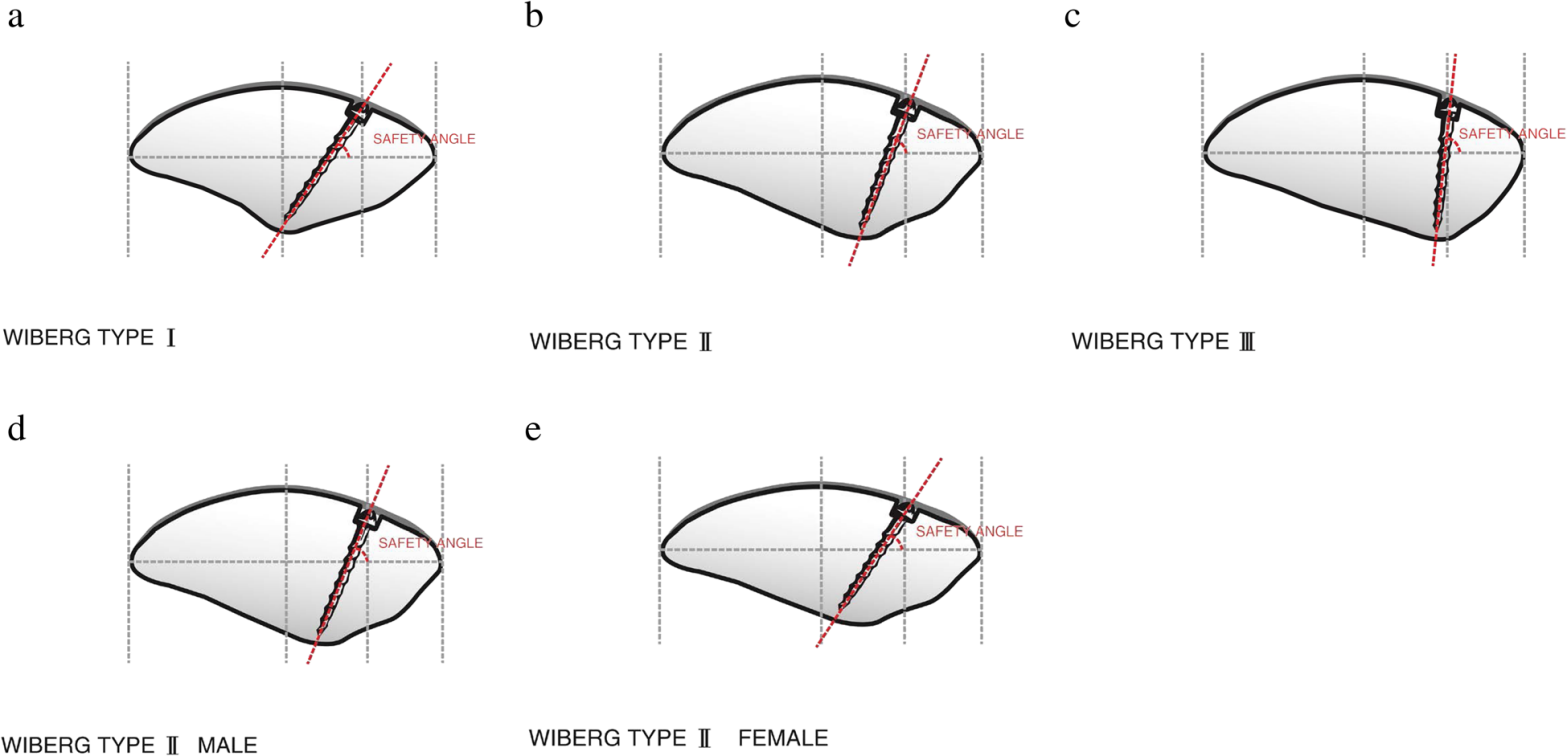
**a**, **b**, **c** Safety angle according to Wiberg type classification. Safety angle becomes more and more vertical from type I to type III. **d**, **e** The difference between males’ and females’ safety angle. Males’ safety angle is larger than females

Some authors have reported on the direction of the suture or drilling tunnel placement, but all are based on surgeon experience [9, 17]. Our suture anchor placement was also initially based on experience, so patellar perforation occurred in several cases (Fig. 5). Since the morphology of the patellar is different, the drilling direction of the suture anchor varies. The thickness at the median ridge was on average 24.7 mm (SD 2.5) [22]. The total length of the suture anchor and bone groove is 17.5 mm. Therefore, to prevent the suture anchor from passing though the patella and to avoid articular cartilage injury, it appears that the safety angle plays a necessary role in guiding the suture anchor into the patella. As the medial patellar articular surface becomes shorter and shorter, and the lateral articular surface longer and longer according to the patellar Wiberg type classification, the thickest part of the patellar is closer and closer to the medial border of the patella, so the safety angle of placing the suture anchor should be increased (Fig. 6a–c and Table 3). Males’ patella are wider and thicker than females’, so we found that the males’ safety angle is larger than females’ (Fig. 6d, e). Comparing with patients with other types of patellar and male patients, we should pay more attention to patients with type I and female patients during fixing of the suture anchor. Additionally, we should take error into consideration and recommend a fixing angle smaller than the safety angle, in case of creating the bone groove too deep. Once the fixing angle is larger than the safety angle, it is easy to injure the patellofemoral joint. Therefore, evaluating the morphology of the patellar and creating the bone groove accurately can decrease the risk of the patellofemoral joint. The safety angle provides a reliable guide for placing the suture anchor.

**Table 3:** The safety angle of different morphology patellar

		Male	Female	*P* value
Type I	Case	13	16	
	Safety angle	45.32 ± 1.76	41.20 ± 1.33	0.01
Type 2	Case	27	38	
	Safety angle	69.74 ± 1.38	63.66 ± 1.45	0.02
Type 3	Case	10	13	
	Safety angle	84.11 ± 1.67	80.26 ± 1.73	<0.01

Although we previously fixed the graft to the patella using a bone groove alone and achieved satisfactory clinical outcomes, the patella rotated and recurrent dislocations occurred during normal flexion-extension movement within months of reconstruction. Some studies have discussed the influence of tendon-bone interface pressure on tendon biology, especially with respect to anterior cruciate ligament (ACL) reconstruction [31, 32]. Park et al. [33] hypothesized that stronger and faster tendon-bone healing may be expected at repaired tendon-bone interfaces with optimal pressure distribution. We propose that the micro-motion of the graft in the bone groove occurs during knee flexion-extension, increasing the risk of delayed or insufficient tendon-to-bone healing. This results in early graft loosening or slackening. To avoid this, we used suture anchors to fix the graft into the bone groove, and the tightly sutured deep fascia with the graft as performed in our technique provides a possible pressure distribution at the tendon-bone interface.

Anatomy is crucial to the success of MPFL reconstructions. The benefit of anatomic graft positioning in ligament reconstruction has been known for a long time and has been clearly demonstrated in ACL reconstructions. Many anatomic studies have illustrated the insertion of the MPFL on the upper two thirds to upper one half of the medial margin of the patella [22, 34, 35]. Placella et al. [22] reported that the MPFL goes from the patella to the femur and has a sail-like shape. The medial third of the patella body is involved in the insertion. The proximal third of the patella is always included in the MPFL attachment. The length of the patellar insertion is an average of 24.5 mm. Parker et al. compared the patellofemoral kinematics of a single-stranded isometric MPFL reconstruction with that of a double-stranded anatomic technique that more closely recreates the anatomy of the MPFL [36]. These studies provide an anatomic basis for anatomical reconstruction, including anatomical insertion, double-bundle structure and sail-like shape of the graft in our technique. Amis et al. [37] reported that the mean load to failure force of the MPFL is 208 N. Some biomechanical studies have demonstrated that the failure load of fixation with suture anchor is larger than 208 N. Lenschow et al. [38] and Hapa et al. [39] reported the failures are 401.5 and 299 N, respectively. Also, the bone groove and deep fascia can strengthen the suture anchor and reduce the micro-motion of the graft. So this fixation can restore the biomechanics of MPFL.

### Limitations

Several limitations to our study must be considered. Our sample size is small and does not include a control group for comparison. We will add a larger number of cases and a comparison group in further studies. Furthermore, although we achieved satisfactory clinical outcomes during our follow-up period, there were still some potential complications of this procedure. First, the risk of vertical fracture of the patella after minor trauma; second, inadequate graft fixation with only one suture anchor, which needs biomechanical studies and more clinical experience. Finally, bulging of the anterior patellar may cause discomfort such as during squatting or kneeling.

## Conclusions

We achieved encouraging results with a patella fixation technique that used a bone groove combined with a suture anchor for patients with recurrent patella dislocations. The safety angle of drilling the suture anchor is not a content according to different morphology of the patellar. When drilling from Wiberg type I to type III patellar, the suture anchor should be more vertical. When fixing the patellar of female patients, the drilling suture anchor should be more sloping. Therefore, we should pay special attention when drilling the suture anchor on type I and female patients. The safe angle provides surgeons with a safe and easy guide for MPFL reconstruction. We believe that this technique may become a popular surgical technique for MPFL reconstruction following further validation.

## Abbreviations

MPFL: Medial patellofemoral ligament
PACS: Picture archiving and communications system
PBT: Patellar bone tunnel
PSA: Patellar suture anchor
